# Association of short-term exposure to ambient carbon monoxide with hospital admissions in China

**DOI:** 10.1038/s41598-018-31434-1

**Published:** 2018-09-06

**Authors:** Hui Liu, Yaohua Tian, Xiao Xiang, Man Li, Yao Wu, Yaying Cao, Juan Juan, Jing Song, Tao Wu, Yonghua Hu

**Affiliations:** 1 Department of Epidemiology and Biostatistics, School of Public Health, Peking University, No.38 Xueyuan Road, 100191 Beijing, China; 2 Medical Informatics Center, Peking University, No.38 Xueyuan Road, 100191 Beijing, China

## Abstract

Evidence on the acute effects of ambient carbon monoxide (CO) pollution on morbidity risk in developing countries is scarce and inconsistent. We conducted a multicity case-crossover study in 26 largest cities in China from January, 2014 to December, 2015 to examine the association between short-term exposure to CO and daily hospital admission. We fitted conditional logistic regression to obtain effect estimates of the associations. We also performed subset analyses to explore the health effects of CO at low levels. During the study period, a total of 14,569,622, 2,008,786 and 916,388 all-cause, cardiovascular and respiratory admissions were identified, respectively. A 1 mg/m^3^ increase in the CO concentrations corresponded to a 3.75% (95% CI, 3.63–3.87%), 4.39% (95% CI, 4.07–4.70%), and 4.44% (95% CI, 3.97–4.92%) increase in all-cause, cardiovascular, and respiratory admissions on the same day, respectively. The associations were robust to controlling for criteria co-pollutants. In subset analyses, negative effects of short-term CO exposure on hospital admission were observed at lower concentrations (<1 mg/m^3^), while positive effects were observed at higher concentrations (>2 mg/m^3^). In conclusion, current CO levels in China were significantly associated with increased daily hospital admissions.

## Introduction

Ambient carbon monoxide (CO) is a colorless, odorless, and tasteless air pollutant that is primarily produced by incomplete combustion of carbonaceous fuels such as motor vehicle emission. Epidemiological studies have examined associations between short-term exposure to CO and morbidity and mortality risk, but the results have been mixed^1–7^. The majority of these studies were carried out in western developed countries, with only a few conducted in developing countries^8^. A recent meta-analysis reported that there were only 5 studies that have examined the association of CO with mortality and 5 studies on morbidity in low and middle income countries^8^. Moreover, recent studies reported acute protective effects of CO exposure on cardiovascular and respiratory health^5,6,9^. Considering the differences in characteristics of ambient air pollution (e.g. the level, chemical composition and source of air pollution), weather patterns, and socio-demographic status between developed and developing counties, there remains a need for scientific data specific to developing countries.

China, the largest developing county, has been experiencing rapid economic evolution and urbanization. As a result, ambient CO pollution has become a serious environmental issue in Chinese cities, especially in large cities where the number of motor vehicles has increased rapidly over the past few decades. Several local studies have assessed the acute effects of CO on mortality risk in China^3,4^. A recent national investigation provided further evidence supporting the coherence and plausibility of the short-term association between CO and mortality^7^. However, morbidity risk has been rarely studied in relation to CO in China, due to the lack of morbidity data. Data on the association between CO and morbidity risk in the Chinese population are still inadequate, especially in multicity assessments. Hospital admission data has been widely used as a measure of morbidity outcome in studies evaluating potential health effects of air pollution^10^. Hospital admissions encompass a broader scope of health effects than more grave events such as mortality. For a geographically defined population, hospital admissions greatly outnumber death events, thus having greater statistical power to detect the health effects of air pollution.

Currently, the ambient CO levels in China are well below the current Chinese Ambient Air Quality Standards (CAAQS) (4 mg/m^3^ for 24-h average) in almost all cities^7^. However, to our knowledge, no studies have been done to directly assess the health effects of short-term exposure to these low CO levels. Evidence on the health effects of exposure levels below the current CAAQS is needed for the revision of standard. Therefore, we conducted a multicity study from January 2014 to December 2015 to examine the associations of CO with all-cause, cardiovascular and respiratory hospital admissions in 26 largest cities in China, as well as to explore the health risk at low levels.

## Methods

### Health data

We obtained hospital admission data from electronic hospitalization summary reports of the top ranked hospitals in 26 largest cities in China during the period 2014–2015. The 26 cites covers all the 4 municipalities (Beijing, Tianjin, Shanghai, and Chongqing), 21 of 28 provincial capital cities, and Dalian city, shown in Figure S1. Details on the health database have been previously reported^11,12^. For each hospital admission, data was extracted on the date of admission, age, sex, and primary discharge diagnosis. The primary discharge diagnoses were coded by the International Classification of Diseases, 10th Revision (ICD-10). In this study, we collected data on daily counts of all-cause, respiratory (ICD-10 codes: J00–J99), and cardiovascular (ICD-10 codes: I00–I99) hospital admissions in each city. This study was carried out in accordance with the Declaration of Helsinki and STROBE guidance. Because the data used for this study was collected for administrative purpose without any individual identifiers, this study was exempted from Institutional Review Board approval by the Ethics Committee of Peking University Health Science Center, Beijing, China. The need for informed consent was also waived by the Institutional Review Board.

### Environmental data

We obtained data on daily CO concentrations in each city from the National Air Quality Real-time Publishing Platform. The platform began operation in 2013, and was administrated by the China’s Ministry of Environmental Protection. There are 4 to 15 monitors in each city. The Chinese government has issued a series of regulations and standards for the selection of monitors’ locations and air quality monitoring process^13^. Prior studies have demonstrated that the measurements could represent general urban background air pollutants levels^14,15^. The monitoring data have been widely used to represent population exposure in studies evaluating the health impacts of air pollution in China^15–17^. We also collected data on daily average concentrations of PM_2.5_ (particles with an aerodynamic diameter ≤2.5 μm), PM_10_ (particles with an aerodynamic diameter ≤10 μm), nitrogen dioxide (NO_2_), sulfur dioxide (SO_2_), and maximum 8-hour mean ozone (O_3_) concentrations from the same platform. Meteorological variables, including air temperature and relative humidity, were obtained from the China Meteorological Data Sharing Service System (http://data.cma.cn/).

### Study design

We examined the short-term associations between CO exposure and daily hospital admission using a time-stratified case-crossover design^18^. For the same person, CO concentration on the day of admission was compared with CO concentrations on other days on the same day of the week within the same month^18^. This design effectively controls the confounding effects of day of the week, time-trends, and confounders that remain constant within a month (e.g., age, sex, and genetics).

### Statistical analysis

Following an approach applied in previous studies^11,12,19^, we performed pooled analyses, for which observations for all cities were combined. A special indicator was assigned to each city in the dataset. We fitted conditional logistic regression to obtain the odds ratio (OR) of hospital admissions associated with short-term exposure to CO following a method used in previous studies. Natural cubic splines of air temperature and relative humidity with 3 degrees of freedom were included in the regression model to control for the confounding effects of weather. The selection of degrees of freedom was based on the parameters used in previous studies^11,20^. We also incorporated interaction terms between cities and air temperature and relative humidity to accommodate potential residual confounding by spatial variations in weather effects. An indicator of public holiday was also included in the model.

We examined the associations between CO and hospital admission with both single-day lags (lag0 to lag5) and successive-day lags (lag0–2 and lag0–5). To explore the potential modifiers of the associations, we did subgroup analyses by sex (male and female) and age (0–17, 18–64, 65–74, and ≥75 years). *P*-values for differences between subgroups were calculated using Z-tests^21^. We also fitted two-pollutant models with adjustment of other criteria air pollutants (PM_2.5_, PM_10_, NO_2_, SO_2_, and O_3_). All the variables (temperature, relative humidity and public holiday) included in single-pollutant models were also incorporated in two-pollutant models.

To assess the associations at low CO concentrations, we conducted subset analyses that only includes days with daily CO concentrations below a specified value. Considering the sample size, we performed the subset analyses for values from 1 to 4 mg/m^3^ in 0.5 mg/m^3^ increments. The restrictions were only applied to the ‘case days’.

All analyses were carried out using R, version V.3.2.2 (R Foundation for Statistical Computing, Vienna, Austria). Effect estimates were presented as percentage changes and 95% confidence intervals (CIs) in daily hospital admissions in relation to per 1 mg/m^3^ increase in daily CO concentrations.

## Results

Table 1 summarizes the demographic characteristics of all-cause, cardiovascular and respiratory admissions from 26 cities in China during 2014–2015. A total of 14,569,622, 2,008,786 and 916,388 admissions for all-cause, cardiovascular, and respiratory diseases were analyzed in this study. The sex and age distributions varied by health outcomes. Table 2 shows summary statistics of air pollutants and weather conditions. The overall mean daily CO concentration was 1.15 mg/m^3^, well below the current CAAQS (4 mg/m^3^). The daily CO concentrations were moderately and positively correlated with PM_2.5_, PM_10_, NO_2_, SO_2_ (correlation coefficient r = 0.55–0.68), while were negatively and weakly correlated with O_3_ (r = −0.26) (Table S1).

**Table 1:** Demographic characteristics of all-cause, cardiovascular and respiratory admissions in 26 cities in China during 2014–2015.

Variable	All-cause admissions	Cardiovascular admissions (ICD-10: I00–J99)	Respiratory admissions (ICD-10: J00–I99)
Total	14,569,622	2,008,786	916,388
Sex			
Male (%)	7,089,060 (48.7)	1,204,769 (60.0)	550,573 (60.1)
Female (%)	7,480,562 (41.3)	804,017 (40.0)	365,815 (39.9)
Age (year) (mean ± SD)	46.9 ± 22.1	60.1 ± 15.6	41.3 ± 30.6
0–17 (%)	1,716,553 (11.8)	33,210 (1.7)	282,274 (30.8)
18–64 (%)	9,704,635 (66.6)	1,166,427 (58.1)	388,021 (42.3)
65–74 (%)	1,919,472 (13.2)	449,636 (22.4)	105,425 (11.5)
≥75 (%)	1,228,962 (8.4)	359,513 (17.9)	140,668 (15.4)

**Table 2:** Summary statistics for air pollutants concentrations and meteorological variables in 26 cities in China during 2014–2015.

Variable	Mean ± SD	Minimum	Percentile			Maximum
			25th	50th	75th	
CO (mg/m^3^)	1.15 ± 0.63	0.14	0.76	0.99	1.32	8.41
PM_2.5_ (μg/m^3^)	63.5 ± 50.6	5.1	31.5	49.4	79.0	897.5
PM_10_ (μg/m^3^)	106.8 ± 71.9	7.4	58.3	89.4	135.2	977.3
NO_2_ (μg/m^3^)	44.1 ± 19.4	4.5	30.0	40.2	54.1	175.8
SO_2_ (μg/m^3^)	29.6 ± 32.6	1.9	11.4	18.8	33.6	316.9
O_3_ (mg/m^3^)	97.4 ± 53.9	2	58	87	129	357
Temperature (°C)	14.5 ± 10.9	−25.7	7.0	16.4	23.3	35.5
Relative humidity (%)	69.2 ± 33.2	8	53	69	80	97

CO = carbon monoxide; PM_2.5_ = particulate matter with aerodynamic diameter < 2.5 μm; PM_10_ = particulate matter with aerodynamic diameter < 10 μm; NO_2_ = nitrogen dioxide; SO_2_ = sulfur dioxide; O_3_ = ozone.

**Figure 1:**
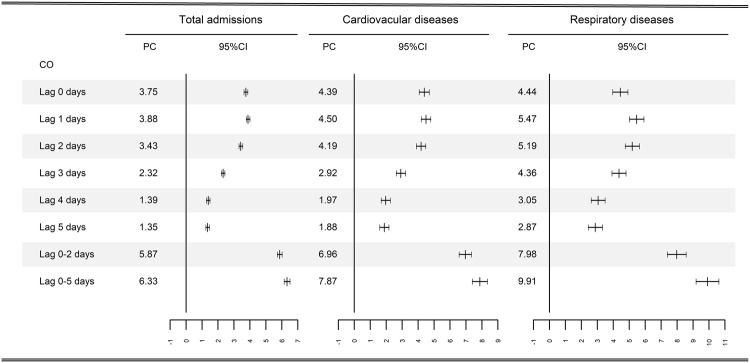
Percentage change (PC) with 95% confidence interval (CI) in hospital admissions associated with 1 mg/m^3^ increase in daily carbon monoxide concentrations for different lag structures.

Figure 1 presents the percentage changes in all-cause, cardiovascular and respiratory admissions associated with a 1 mg/m^3^ increases in CO concentrations for different lag structures. A 1 mg/m^3^ increase in the same-day CO concentrations was significantly associated with a 3.75% (95% CI, 3.63–3.87%), 4.39% (95% CI, 4.07–4.70%), and 4.44% (95% CI, 3.97–4.92%) increase in all-cause, cardiovascular and respiratory admissions, respectively. Table 3 shows the results of two-pollutant models. The effect estimates increased after adjustment of PM_2.5_, PM_10_, SO_2_, and O_3_; these estimates decreased after controlling for NO_2_.

**Table 3:** Percentage changes with 95% confidence intervals in admissions associated with 1 mg/m^3^ increases in same-day carbon monoxide concentration in 2-pollutant models.

Variable	All-cause admissions	Cardiovascular admissions	Respiratory admissions
Adjust PM_2.5_	4.93 (4.75, 5.10)	5.61 (5.16, 6.05)	5.16 (4.48, 5.84)
Adjust PM_10_	4.88 (4.71, 5.04)	5.37 (4.95, 5.79)	4.63 (3.99, 5.26)
Adjust NO_2_	2.16 (2.00, 2.32)	3.10 (2.68, 3.52)	2.71 (2.08, 3.35)
Adjust SO_2_	7.23 (7.08, 7.38)	7.99 (7.62, 8.36)	9.23 (8.66, 9.80)
Adjust O_3_	3.82 (3.70, 3.94)	4.44 (4.13, 4.75)	4.50 (4.03, 4.98)

PM_2.5_ = particulate matter with aerodynamic diameter < 2.5 μm; PM_10_ = particulate matter with aerodynamic diameter < 10 μm; NO_2_ = nitrogen dioxide; SO_2_ = sulfur dioxide; O_3_ = ozone.

Figure S2 shows the exposure-response relationship curves of carbon monoxide concentrations and daily total hospital admissions. There was a concentration-dependent association with negative association at low levels (<1 mg/m^3^) and positive associations at high levels (>1 mg/m^3^).

**Figure 2:**
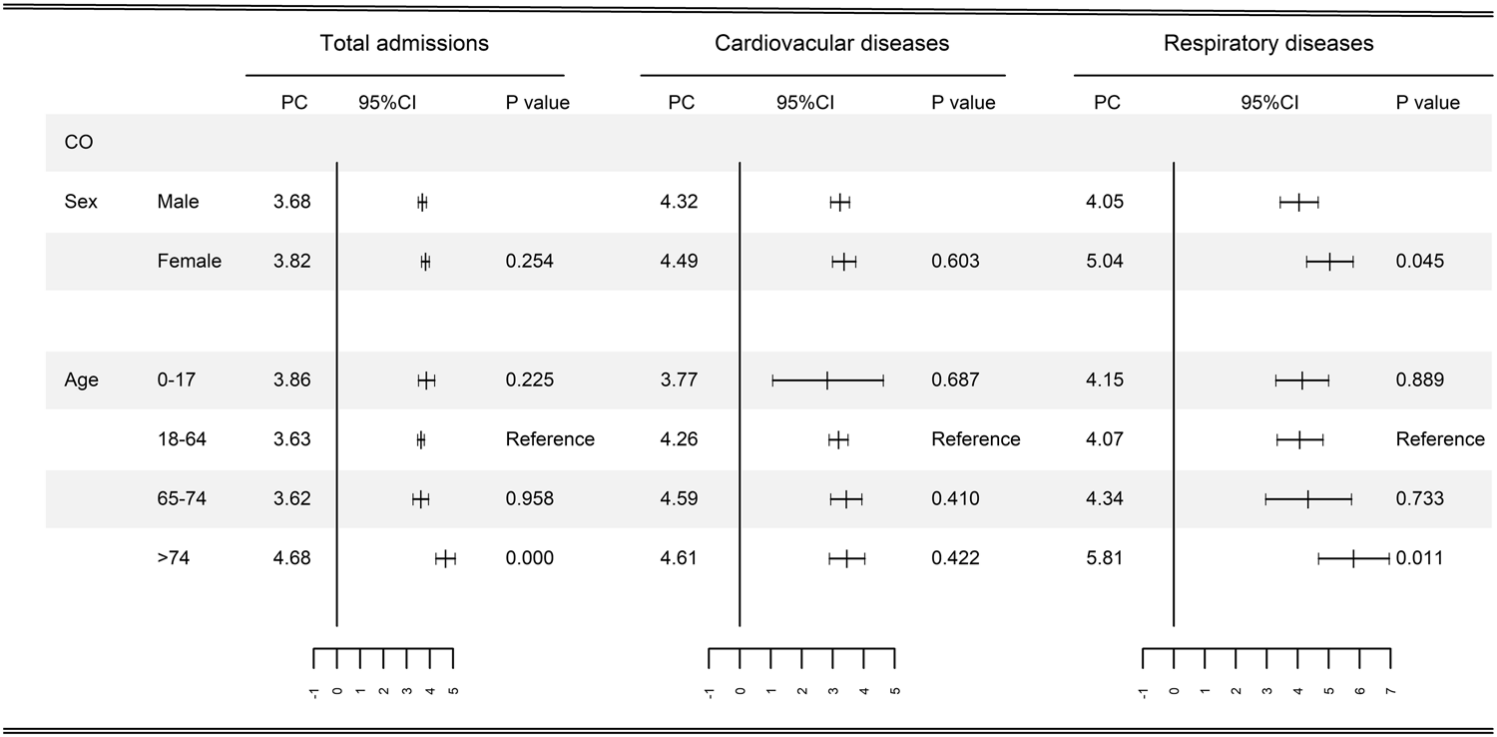
Percentage change (PC) with 95% confidence interval (CI) in hospital admissions associated with 1 mg/m^3^ increase in daily carbon monoxide concentrations, classified by age and sex. *P*-value obtained from Z test for the difference between the two risk estimates derived from subgroup analysis.

The associations between short-term exposure to CO (lag 0) and hospital admissions varied by age and sex (Fig. 2). We consistently observed larger effect estimates in females than in males among all-cause, cardiovascular, and respiratory admissions, but the differences between subgroups were only statistically significant among respiratory admissions. We also observed stronger effects in people aged ≥75 years among all the three causes of admissions, but the difference between subgroups was not significant among cardiovascular admissions.

**Figure 3:**
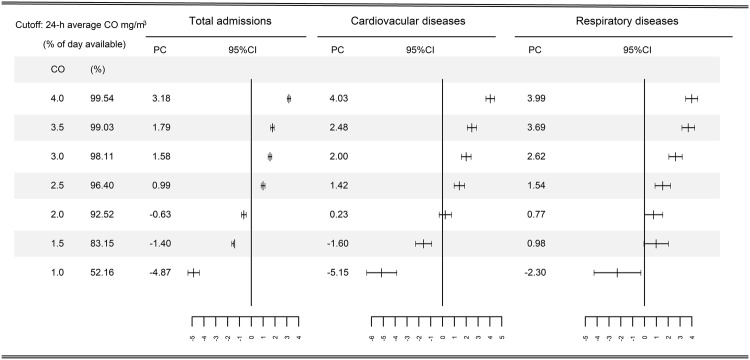
Percentage change (PC) with 95% confidence interval (CI) in hospital admissions associated with 1 mg/m^3^ increase in daily carbon monoxide concentrations based on the subset of data only including days with concentrations below the specified values specified on the X-axis. The numbers in second column indicate the percent of days used in each threshold analysis, on average across the cities, compared to the data used in the non-threshold analysis.

Figure 3 presents the results of the subset analyses. We observed similar concentration-dependent patterns among all-cause, cardiovascular, and respiratory admissions. Short-term exposure to CO was positively associated with daily hospital admission at higher concentrations (>2 mg/m^3^), while it showed negative association at lower levels (<1 mg/m^3^).

## Discussion

In this multicity study in 26 largest cities in China covering >14 million hospitalizations, short-term exposure to CO was significantly associated with daily hospital admissions for all-cause, cardiovascular, and respiratory diseases. The associations were robust to the adjustment of co-pollutants (PM_2.5_, PM_10_, NO_2_, SO_2_, and O_3_). The associations appear to be more evident in females and elderly. In subset analyses, we observed negative effects at lower levels (<1 mg/m^3^), and positive effects at higher levels (>2 mg/m^3^). To our knowledge, this is the first multicity study in China to report significant effects of CO on morbidity risk.

Overall, we found a positive association between short-term exposure to CO and hospital admission for cardiovascular diseases. Our results were generally consistent with previous multicity and meta analyses worldwide. For example, Bell *et al*.^2^ estimated that 1 ppm (≈1.25 mg/m^3^) increase in same-day daily 1-h maximum CO concentrations was associated with a 0.96% (95% CI, 0.79%, 1.12%) increase in emergency hospital admissions for cardiovascular diseases among Medicare enrollees aged ≥65 years in 126 U.S. urban counties between 1999 and 2005. In line with the findings of this U.S. study, we found that the association remained statistically significant with co-pollutant adjustment. Similarly, a case-crossover study in 7 Australian and New Zealand cities found that 0.9 ppm increase in daily CO concentrations corresponded to a 2.2% (95% CI, 0. 9%, 3.4%) increase in hospitalizations for cardiovascular diseases^22^. Also, a recent meta-analysis demonstrated a significant association between short-term exposure to CO and cardiovascular admissions^23^. The broad consistency in the literature suggests that the association between short-term exposure to CO and cardiovascular hospital admission is unlikely to be spurious because of publication bias, confounding, or flaws in study design.

The acute effects of CO exposure on human respiratory health have seldom been explored in population-based epidemiological studies. The U.S. Environmental Protection Agency have demonstrated that evidence on the effects of CO on respiratory outcomes have been limited and inconclusive^5^. The inconsistency in the effect estimates may be attributable to variations in CO levels. In this study, we observed a negative association between CO and daily hospital admissions for respiratory diseases at lower levels (<1 mg/m^3^), and a positive association at higher levels (>2 mg/m^3^). Two time-series studies done in Hong Kong from January 2001 to December 2007 found that short-term exposure to CO was negatively associated with the risk of hospital admissions for respiratory tract infection and chronic obstructive pulmonary disease^5,6^. The daily average CO concentration during the study period was 0.6 ppm (≈0.75 mg/m^3^). These findings were consistent with the negative effects of CO at lower levels (<1 mg/m^3^) observed in our study. A study conducted in Guangzhou, Foshan, Zhuhai, and Zhongshan in the Pearl River Delta of southern China reported mixed results on the associations between CO and daily respiratory mortality, with positive associations observed in Guangzhou and Foshan which have relatively higher CO levels^3^. However, another study conducted in three Chinese cities (Shanghai, Anshan and Taiyuan) reported no association between CO and respiratory mortality^4^. Differing health outcomes, sample sizes, and characteristics of study locations across studies may also affect estimates.

In this study, we consistently observed negative effects of CO on all-cause, cardiovascular, and respiratory hospital admissions at lower levels, and positive effects at higher levels, which is complemented by the exposure-response curve. Our findings were consistent with two local epidemiological studies in China. Both of the two studies found that the values of relative risk for the associations between CO and total/cardiovascular/respiratory mortality were less than 1 at low concentrations (<1.5 mg/m^3^)^3,4^. Moreover, a recent national study in China explored the exposure-response association between CO and mortality, and found that the relative changes in daily mortality were less than 0 at low levels (<1.5 mg/m^3^). Our findings were also supported by experimental studies demonstrating that inhaled CO at low levels may have beneficial anti-inflammatory and anti-microbial effects. CO administered exogenously via CO-releasing molecules could kill bacteria^24^. Inhaled CO could lead to preservation of organ function and improved survival of rodents previously treated with endotoxin^25^. It has also been suggested that CO could strengthen the inflammatory response in common infections^26^. As this was the first population-based study to report mixed concentration-dependent health effects of CO, future studies are warranted to confirm our findings.

The present CAAQS for CO was originally promulgated in 1982. However, to our knowledge, no studies have been done to directly assess the health effects of CO exposure at levels below the current CAQQS. In this study, the overall mean daily CO concentration (1.15 mg/m^3^) was much lower than the current CAAQS (4 mg/m^3^), yet we still observed positive effects of short-term CO exposure on daily hospital admissions. Moreover, when restricting the analyses to daily CO levels at 2.5–4 mg/m^3^, the associations between short-term exposure to CO and hospital admission remained significant. Our findings were consistent with epidemiological evidence from both China and other nations^1,7,27^. These results indicated that current CAAQS for CO could not provide sufficient protection from hazardous impacts of CO exposure in China, and maybe a more stringent air quality standard ought to be considered.

The effects of CO exposure might be unevenly spread throughout populations. Identification of potentially susceptible subpopulations has significant public health implications for air quality standards setting and targeted intervention. Consistent with most previous studies^3,7^, we consistently found that the associations between CO and daily hospital admission for all-cause, cardiovascular, and respiratory diseases appeared to be stronger in females and elderly. Changes in the structure of respiratory system and declined biological function and anti-infection ability may explain the higher effect estimates in the elderly^28,29^. The sex difference in the magnitude of effect estimates may be attributable to hormones and structural/morphological differences in the respiratory system between males and females^30^.

Our study has several limitations. First, as in other time-series studies, we used fixed monitoring measurements as the proxy of population exposure. The resulting exposure measurement error generally underestimates CO effects^31^. Second, we fitted two-pollutant models to examine the robustness of the associations. However, the notable collinearity between CO and other air pollutants made it difficult to precisely evaluate the independent contributions of CO to the admission risk.

In conclusion, we observed significant associations between short-term exposure to CO and increased daily hospital admissions in 26 largest cities in China. Short-term exposure to CO showed concentration-dependent associations with hospital admissions, with negative associations at lower concentrations and positive associations at higher concentrations. Future studies are warranted to confirm our findings and to explore potential biological mechanisms.

## Electronic supplementary material

Supplementary Information
