# Supplementary material for: Association of short-term exposure to ambient carbon monoxide with hospital admissions in China
Source: Sci Rep. 2018 Sep 6;8:13336. doi: 10.1038/s41598-018-31434-1 (PMC6127141; doi:10.1038/s41598-018-31434-1)
Supplement: Supplementary file 1 — Supplementary Information [file 41598_2018_31434_MOESM1_ESM.docx]

**Association of short-term exposure to ambient carbon monoxide with hospital admissions in China**

Hui Liu,^1,2^ Yaohua Tian,^1^ Xiao Xiang,^1^ Man Li,^1^ Yao Wu,^1^ Yaying Cao,^1^ Juan Juan,^1^ Jing Song,^1^ Tao Wu,^1^ and Yonghua Hu^1^

^1^ Department of Epidemiology and Biostatistics, School of Public Health, Peking University, No.38 Xueyuan Road, 100191 Beijing, China

^2^ Medical Informatics Center, Peking University, No.38 Xueyuan Road, 100191 Beijing, China

**Hui Liu and Yaohua Tian contributed equally to the manuscript and should be considered as co–first authors.**

**Yonghua Hu and Tao Wu contributed equally to the manuscript and should be considered as co–corresponding authors.**

**Corresponding author:** Yonghua Hu Prof., M.D, Department of Epidemiology and Biostatistics, School of Public Health, Peking University, No.38 Xueyuan Road, 100191 Beijing, China, Tel.: +86-135-0136-1139, Fax: +86-10-82801189, E-mail: [yhhu@bjmu.edu.cn](mailto:yhhu@bjmu.edu.cn); Tao Wu Prof., Ph.D., Department of Epidemiology and Biostatistics, School of Public Health, Peking University, No.38 Xueyuan Road, 100191 Beijing, China, Tel.: +86-139-1003-3458, Fax: +86-10-82801189, E-mail: twu@bjmu.edu.cn

**Table S1.** Spearman correlation coefficients among the exposure variables.

| Variables | CO | PM_2.5_ | PM_10_ | NO_2_ | SO_2_ | O_3_ | Temp | RH |
| --- | --- | --- | --- | --- | --- | --- | --- | --- |
| CO | 1.00 | 0.68^a^ | 0.60^a^ | 0.59^a^ | 0.55^a^ | -0.26^a^ | -0.37^a^ | 0.02^b^ |
| PM_2.5_ | ― | 1.00 | 0.87^a^ | 0.67^a^ | 0.61^a^ | -0.06^a^ | -0.32^a^ | -0.08^a^ |
| PM_10_ | ― | ― | 1.00^a^ | 0.64^a^ | 0.63^a^ | 0.01 | -0.27^a^ | -0.33^a^ |
| NO_2_ | ― | ― | ― | 1.00 | 0.54^a^ | -0.13^a^ | -0.36^a^ | -0.11^a^ |
| SO_2_ | ― | ― | ― | ― | 1.00 | -0.18^a^ | -0.51^a^ | -0.36^a^ |
| O_3_ | ― | ― | ― | ― | ― | 1.00 | 0.64^a^ | -0.15^a^ |
| Temp | ― | ― | ― | ― | ― | ― | 1.00 | 0.18^a^ |
| RH | ― | ― | ― | ― | ― | ― | ― | 1.00 |

^a^*P* < 0.001

^b^*P* < 0.05

CO = carbon monoxide; PM_2.5_ = particulate matter with aerodynamic diameter <2.5 μm; PM_10_ = particulate matter with aerodynamic diameter <10 μm; NO_2_ = nitrogen dioxide; SO_2_ = sulfur dioxide; O_3_ = ozone; Temp = temperature; RH = relative humidity.**
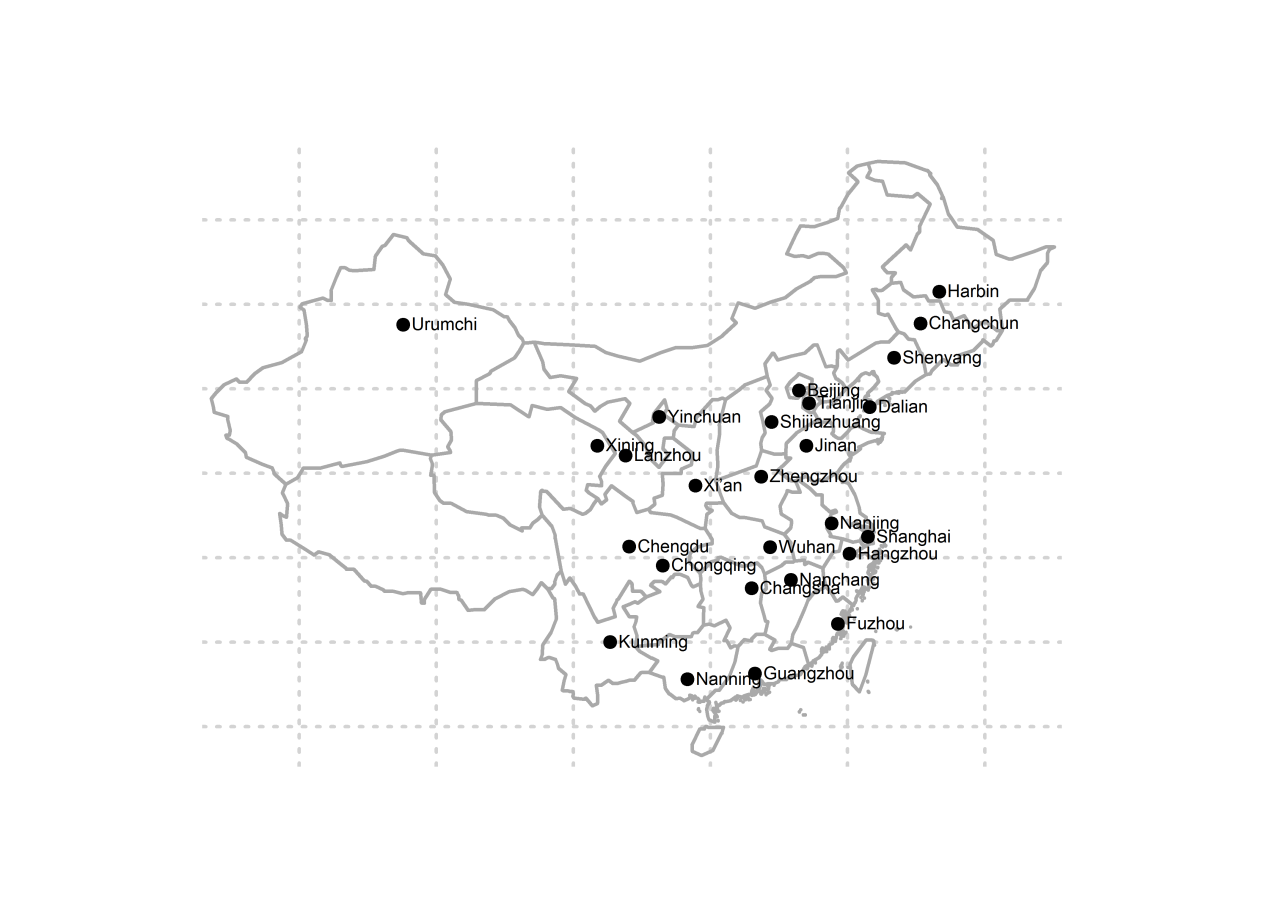
**

**Figure S1.** Locations of the 26 cities in China in this study.


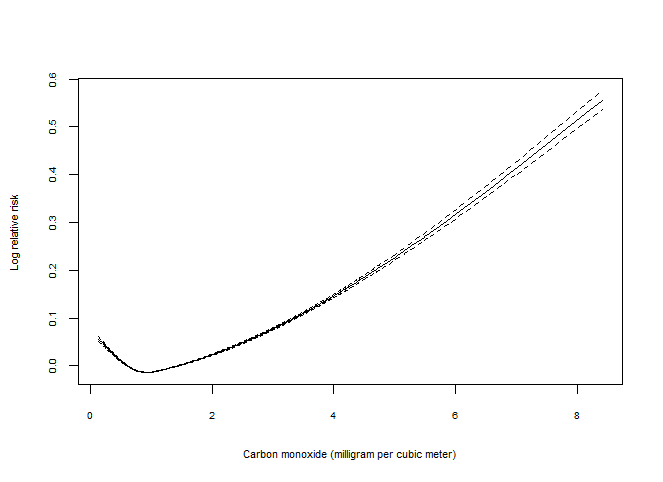


**Figure S2.** The exposure-response relationship curves of carbon monoxide concentrations and daily total hospital admissions.
